# Macro- and Microelement Content and Other Properties of *Chaenomeles japonica* L. Fruit and Protective Effects of Its Aqueous Extract on Hepatocyte Metabolism

**DOI:** 10.1007/s12011-017-0931-4

**Published:** 2017-01-18

**Authors:** Irena Baranowska-Bosiacka, Beata Bosiacka, Julita Rast, Izabela Gutowska, Jolanta Wolska, Ewa Rębacz-Maron, Kamila Dębia, Katarzyna Janda, Jan Korbecki, Dariusz Chlubek

**Affiliations:** 1 Department of Biochemistry and Medical Chemistry, Pomeranian Medical University, Powstańców Wlkp. 72 Str, 70-111 Szczecin, Poland; 2 Department of Plant Taxonomy and Phytogeography, University of Szczecin, Wąska 13 Str, 71-415 Szczecin, Poland; 3 Department of Biochemistry and Human Nutrition, Pomeranian Medical University, Broniewskiego 24 Str, 71-460 Szczecin, Poland; 4 Department of Vertebrate Zoology and Anthropology, University of Szczecin, Wąska 13 Str, 71-415 Szczecin, Poland

**Keywords:** Japanese quince extract, *Chaenomeles japonica* L., Hepatocyte metabolism, Lipid peroxide, Reactive oxygen species (ROS), Apoptosis

## Abstract

This growing interest in the cultivation of Japanese quince *Chaenomeles japonica* L. results from the potentially beneficial properties of its fruit. Fresh fruits are very firm and too acidic to eat raw, but their bioactive components, distinctive aroma, and high amount of dietary fiber make the fruits well suited for industrial processing. However, not all the properties of the fruit have been investigated. For example, there are no comprehensive reports about the mineral content or potentially harmful effects on liver metabolism. Hence, the purpose of our study was to examine fresh Japanese quince fruit in terms of (1) ascorbic acid, oxalate, fiber, macro- and micronutrients, dry matter, extract, total acidity, antioxidant activity, and phenolic compound levels; and (2) the effect of its extract on in vitro hepatocyte metabolism, measured by the concentration of lipid peroxides (LPO) and reactive oxygen species (ROS) and the severity of apoptosis and necrosis. The fruit of *C. japonica* had high levels of macro- and microelements, ascorbic acid, phenolic compounds, fiber, and low oxalate levels. Our analysis of macro- and microelements showed that the average content of Fe was 0.516 mg/g, Cu 0.146 mg/g, Zn 0.546 mg/g, Mg 16.729 mg/g, and Ca 22.920 mg/g of fresh fruit. A characteristic feature of the fresh fruit of *C. japonica* is a high level of polyphenols, which—combined with a high content of vitamin C—affect their high antioxidant potential. In the tested hepatocyte cultures incubated with extract of the Japanese quince, we observed a significant decrease in the concentration of lipid peroxides compared to the control. There were also no signs of increased formation of ROS in the mitochondria of hepatocytes incubated with the extract of quince. Malondialdehyde was strongly negatively correlated with the concentration of Japanese quince extract, which indicates the hepatoprotective properties of Japanese quince. In addition, our analysis of confocal microscopy images showed that the hepatocytes incubated with the extract of Japanese quince at any concentration did not show any signs of apoptosis or necrosis. The aqueous extract of quince fruit has antioxidative and antiapoptotic hepatocytes, thus exerting a hepatoprotective effect.

## Introduction

Japanese quince (*Chaenomeles japonica*), a short shrub naturally occurs in central and southern Japan, is one of four species belonging to the genus *Chaenomeles*, family Rosaceae [18]. Compared to the other three species (*Chaenomeles cathayensis*, *Chaenomeles speciosa*, and *Chaenomeles thibetica*), *C. japonica* is best suited to the climate of northern Europe, where it was introduced in 1869 [39]. Research on its usefulness and growing conditions has been carried out in Poland [10, 12], Ukraine [14], Lithuania [22], Finland [35], and Latvia [26]. In 1998, multidisciplinary international research was initiated to examine the potential of *C. japonica* as a new fruit crop for Northern Europe [24, 27].

This growing interest in the cultivation of Japanese quince results from the potentially beneficial properties of its fruit. In East Asia, especially in traditional Chinese medicine, the fruits of various species of *Chaenomeles* have been used for thousands of years in the treatment of rheumatoid arthritis, hepatitis, asthma, and the common cold [44, 45]. It contains many bioactive components, including polyphenols, triterpenes, and organic acids [2, 4, 6, 10, 11, 13, 21, 23, 28]. The most important properties of these components in the fruits of the various *Chaenomeles* species, and other plants include a protective effect against cardiovascular diseases and antitumoral, anti-inflammatory, and antioxidant activities (e.g., [20, 37, 40, 44]).

Fresh *Chaenomeles* fruits are very firm and too acidic to eat raw, but their bioactive components, distinctive aroma, and high amount of dietary fiber make the fruits well suited for industrial processing. They can be used to make juice, wine, syrup, liquor, lemonade, marmalade, and fragrance extract [5, 10, 12, 24, 27, 33, 34]. A study of consumer preferences towards various products with added Japanese quince puree, syrup and jam, showed very positive reactions to the taste [25]. However, not all the properties of the fruit have been investigated. For example, there are no comprehensive reports about the mineral content or potentially harmful effects on liver metabolism. Hence, the purpose of our study was to examine fresh Japanese quince fruit in terms of (1) ascorbic acid, oxalate, fiber, macro- and micronutrients, dry matter, extract, total acidity, antioxidant activity, and phenolic compound levels; and (2) the effect of its extract on in vitro hepatocyte metabolism, measured by the concentration of lipid peroxides (LPO) and reactive oxygen species (ROS) and the severity of apoptosis and necrosis.

## Material and Methods

The study was carried out on commercially available fresh fruit of *C. japonica* (a fruit pulp from the peel, seedless, and seed envelope) and its aqueous extract. All fruits were collected at the same state of ripeness according to the specification of Abbott [1]: when the fruit skin had turned yellow and the seed coat had turned brown, indicating maturity.

## Fresh Fruit Analysis

### l-Ascorbic Acid Determination

One hundred grams of fresh fruit was frozen in liquid nitrogen and stored at −70 °C until analysis. Pulverized samples (0.1 g) were mixed with 5 mL of methylphosphoric acid. The mixture was homogenized for 5 min and then centrifuged at 10000 G for 10 min at +4 °C. The extraction procedure was carried out at 4 °C, and the samples were protected from light. The extracts were stored at +4 °C within 0.5 h of analysis. The samples were analyzed by isocratic HPLC method (Agilent 1050/1100 chromatograph) at +21 °C on a LiChrospher 100 RP-18 125 × 4-mm column (Merck, Germany) using 0.1 M phosphate buffer (pH = 3) as mobile phase. Determination of the l-ascorbic acid was conducted at 243 nm. Quantitation was performed by comparing the chromatographic peak area with that of the external standard. A calibration curve was plotted in the concentration range of 0.5–200 mg/L and based on a ten-point calibration [17]. The recovery value (as a measure of accuracy) was 98%. The RSD value (as a measure of precision) was 9.5%. The LOD value was 0.2 mg/L, and LOQ was 0.5 mg/L.

### Oxalate Determination

0.1 g of fresh fruit homogenate was quenched with 2 mL of 1 M H_2_SO_4_. Then, it was vortexed to see if it released CO_2_. The tubes were then inserted into a water bath (100 °C) for 1 h. When the content was completely dissolved, it was centrifuged and, after cooling, a 1 mL of sodium acetate CaCl_2_ was added to the sample tube, vortexed, and then left for 1 h. The presence of precipitate indicated the presences of oxalate. Then, the content of the sample tube was centrifuged and the supernatant discarded, leaving only the precipitate to which 1 mL of 1 M H_2_SO_4_ was added. The mixture was dissolved in a water bath (100 °C) and titrated hot with 0.1 N KMnO_4_ to a permanent pink coloration [9]. The recovery value of the method was 95%. The RSD value was 10.5%. The LOD value was 0.1 mg/g, and LOQ was 0.25 mg/g.

### Dietary Fiber Determination

Ten samples of fresh fruit (3 g each) were weighed in 100-mL flasks. Then, a mixture of the following acids was prepared: 70% acetic acid—187.5 mL, nitrogen acid—12.5 mL, and trichloroacetic acid (5 g), and 20 mL of the mixture was added to each of the ten samples. Earlier, filters had been dried in the vessels and then weighed. Each of the ten samples was filtered through the previously prepared filters and rinsed with water until no acidity was found. Finally, the samples were rinsed twice with 20 mL of ethyl alcohol and dried to a constant mass [7]. The recovery value of the method was 98%. The RSD value was 12%. The LOD value was 0.03 g, and LOQ was 0.05 g.

### Macro- and Micronutrient Determination

Fruit homogenate, dried to a constant weight each (0.2 g), were weighed in test tubes and placed in a thermoblock. The samples were mineralized in a mixture of 1 mL HClO_4_ and 4 mL of 70% HNO_3_. The samples were heated in a block for 12 h at 120 °C and then at 180 °C for 3 h until total apparent mineralization of samples and drying of the mixture of acids. After the completion of mineralization, the tube content was quantitatively transferred with deionized water to flasks and supplemented with deionized water (Millipore) to a volume of 25 mL [36]. The analysis of elements (Ca, Mg, Zn, Cu, Fe, P, K, Na, Mn, Mo) was performed using atomic absorption spectrophotometry, Solar AAS-969. The LOD values for all studied elements was 0.0002 mg/g, and LOQ was 0.002 mg/g. Recovery and RSD values were for Ca—98, 9.95%; for Mg—99, 8.9%; for Zn—99, 10.5%; for Cu—99.5, 10%; for Fe—100, 9%; P—99.9, 9%; for K—95, 8.5%; for Na—98.5, 8%; for Mn—98.5, 10%; and for Mo—99, 10% appropriately.

### Dry Weight Determination

Homogenized fruit samples were dried in an oven with hot air at a temperature of 105 °C until a constant weight, measured with an analytical balance [8].

### Extract Content Determination

Fruit homogenate (40 g) was supplemented with 160 mL of distilled water and homogenized using a high-speed homogenizer, Ultra-Turrax T 25 basic (5 min, 22,000 rpm). Then, 100 g of homogenate was collected, boiled for 5 min, cooled, supplemented with distilled water to 100 g, and filtered. Extract (%) was determined by refractometry, (Abbe), Donserv (Warsaw, Poland) [29]. The recovery value of the method was 99%. The RSD value was 9.5%. The LOD value was 0.02%, and LOQ was 0.05%.

### Total Acidity Determination

Fruit homogenate (40 g) was supplemented with 160 mL of distilled water and homogenized using an Ultra-Turrax T 25 high-speed basic homogenizer (5 min, 22,000 rpm). Twenty-five-gram samples were weighed in a 250-mL beaker; 100 mL of distilled water was added and heated to boiling point. The solution was then cooled and quantitatively transferred to 250-mL volumetric flasks for 15 min. The content of each flask was filtered; then, 10 mL of the filtrate was dispensed into a conical flask and titrated with 0.1 N NaOH solution with three to four drops of phenolphthalein to a light pink color [19]. The recovery value of the method was 99.5%. The RSD value was 11.5%. The LOD value was 0.005%, and LOQ was 0.01%.

### Determination of Antioxidant Activity by Spectrophotometric Method

ABTS radical was produced in a reaction between 7 mM ammonium salt of the 2,2′-azino-bis(3-ethylbenzothiazoline-6-sulphonic acid) and 2.45 mM potassium metabisulfite. In order to stabilize the ABTS radical, the solution was kept in the dark (at 22–25 °C) for 18 h. The solution was diluted using phosphate buffered saline (PBS) so that the absorbance determined at a wavelength of 734 nm was *A* = 0.70 ± 0.02 (ABTS 0.7). Fruit samples were freeze dried (Christ Ralph 1-4 freeze dryer) and extracted with 80% methanol (0.5 g in 25 mL). The extracts (100 μL) and Trolox solution (concentration 1–10 mg/100 mL) were added to 1 mL of ABTS 0.7, and absorbance was measured at 6 min. Antioxidant activity was determined on the basis of a calibration curve plotted using the synthetic vitamin E (Trolox) and expressed in micromolars of Trolox/100 g of fresh fruit. The recovery value of the method was 100 ± 5%. The RSD value was 9.58%. The LOD value was 0.10 mM Trolox/100 g, and LOQ was 0.20 mM Trolox/100 g.

### Determination of Total Phenolic Compounds

Forty-five milliliters of ultra pure water (Milli-Q, Merck Millipore water purification system, Warsaw, Poland) was added to 0.25 mL of Folin-Ciocalteau (dissolved in water at a ratio 1:1), 0.5 mL of 7% Na_2_CO_3_, and then 5 mL of the fruit extract (0.5 g in 25 mL of 80% methanol). The absorbance of the solution was measured spectrophotometrically (Beckman DU-650, *λ* = 760 nm) after 30 min. The content of phenolic compounds in general was determined on the basis of a calibration curve plotted using catechin and expressed in milligrams of catechin/100 g of fresh fruit [29]. The recovery value of the method was 99%. The RSD value was 10.5%. The LOD value was 1 mg catechins/100 g, and LOQ was 2 mg catechins/100 g.

## In Vitro Studies Using an Aqueous Extract of Japanese Quince

### Preparation of Extract of Japanese Quince

Ten grams of homogenized sample was poured into 100 mL of distilled water at 25 °C. The resulting suspension was placed in a water bath under reflux at 90 °C for 30 min. After this time, the solution was cooled to room temperature and filtered under low pressure. The resulting filtrate was subjected to vacuum distillation to remove the water and obtain a dry solid material.

### In Vitro Hepatocyte Culture

*HepG2* hepatocytes were cultured in Eagle’s minimum essential medium (EMEM, Sigma-Aldrich) supplemented with fetal bovine serum (Gibco); the volume ratio of medium to serum was 9:1. To each milliliter of medium with serum, a 4:1 of mixture of penicillin and streptomycin (Sigma-Aldrich) was added to obtain a complete medium. Cells were placed at a density of one million cells in a 6-hole dish and cultured in an incubator under standard incubation conditions (37 °C and 5% CO_2_). The cells were passaged every 3–4 days when hepatocytes covered 70–80% of the tile.

### Incubation of Hepatocytes with Japanese Quince Extract

Hepatocytes were placed at a density of one million per 6-well plate using complete medium for the control group and a medium enriched with an aqueous extract of quince at final concentrations of 1, 10, 50, and 100 μg/mL. Positive control was a sample prepared with the addition of the medium and 5 μg/mL of dimethyl sulfoxide (DMSO). The initial solution of Japanese quince extract at a concentration of 1 mg/mL was prepared by dissolving concentrated aqueous Japanese quince in DMSO which was then diluted with medium to yield the final concentrations used in the experiment. Cells were incubated for 48 h at standard conditions (temp. 37 °C, 5% CO_2_). After incubation, the cells were scraped from the wells using a rubber scraper, and then the cell suspension was transferred to Falcon on tubes 15 mL type. The cells were centrifuged (4 °C, 700 G, 10 min); the medium from the cells was collected and transferred to separate tubes. The cell pellet was dissolved in 1 mL of PBS at a temp. of 4 °C; 23 μL of samples was taken for protein determination. The cell suspension was centrifuged under conditions as previously, the medium was collected and discarded, and the cell pellet was resuspended in 100 PBS. The cells, samples for protein determination, and collected medium were stored at −80 °C for further analysis.

### Lipid Peroxide (LPO) in Hepatocytes Incubated with Extract of Japanese Quince

In order to determine the concentration of lipid peroxide, hepatocytes incubated with an aqueous extract of the Japanese quince, in concentrations as described previously, were rinsed with PBS (pH 7.4) and immediately immersed in liquid nitrogen. To the thus prepared samples, we added a cold buffer (5 uL) containing chloroform and methanol, and homogenates were made on ice (knife homogenizer CAT X120, Germany). The homogenate was centrifuged at +4 °C for 15 min at 10000 G. After centrifugation, a clear supernatant was obtained, transferred into a new tube, frozen at −80 °C, and used in further analysis.

The level of LPO was determined using a Lipid Hydroperoxide Assay Kit according to the enclosed instruction (Cayman Chemical Company, Lipid Hydroperoxide Assay Kit, no. 705002). The measurement of LPO levels was based on the direct reduction reaction with iron ions. Lipid peroxides are highly unstable and react immediately with iron ions (Fe^3+^) to form ferrous ions (Fe^2+^). By using rhodanide ions as the chromogen, it is possible to determine the level of Fe^2+^. Polyunsaturated fatty acids of cell membranes in the conditions of oxidative stress generate malondialdehyde (MDA) and 4-hydroxy alkenal; thus, the measurement of the MDA concentration can be used to determine lipid peroxidation. The kit used was highly specific to MDA.

### Apoptosis and Necrosis Evaluation in Hepatocytes Incubated with Extract of Japanese Quince

The hepatocytes were cultured on coverslips in 6-well plates under standard conditions by adding the extract to the medium as described previously. Cells incubated with an aqueous extract of the Japanese quince at concentrations as described previously were rinsed with PBS (pH = 7.4) and immediately analyzed for apoptosis using staining with Annexin V and propidium iodide (PI), with the use of the Annexin V-FITC Early Apoptosis Detection Kit. Visualization of apoptosis/necrosis was performed with a Olympus Fluoview 1000 confocal microscope.

### Determination of the Concentration of Reactive Oxygen Species (ROS) in Hepatocytes Incubated with the Japanese Quince Extract

The hepatocytes were cultured on coverslips in 6-well plates under standard conditions by adding the extract to the medium as described previously. Cells incubated with an aqueous extract of Japanese quince in concentrations as described previously were rinsed with PBS (pH = 7.4) and immediately analyzed. The determination of reactive oxygen species (ROS) levels, generated in the mitochondria of hepatocytes, was performed using MitoSOX Red (Invitrogen) superoxide indicator according to the method of Zhang et al., using microplate reader model 680 (Bio-Rad).

To visualize the ROS generated in the mitochondria of the hepatocytes, we used the MitoSOX Red (Invitrogen) according to the method by Zhang et al. [42]. The study was performed using an Olympus Fluoview 1000 confocal microscope.

### Statistical Analysis

The results were statistically analyzed using STATISTICA 10.1 (StatSoft, Poland). For each test parameter, an arithmetic mean (*x̅*) and standard deviation (±SD) were calculated. In order to verify the distribution of the results obtained for the various parameters, we performed Shapiro-Wilk tests. Because most distributions differed from a normal distribution, in further statistical analysis, we used nonparametric tests. In order to determine statistically significant differences for dependent samples, they were first analyzed by Friedman’s ANOVA, and then with Wilcoxon matched pair tests, which assumes that there is an opportunity to give rank magnitudes of differences for paired observations in an unambiguous way. The level of significance was *p* ≤ 0.05.

## Results

### Characteristics of the Japanese Quince Fruit Properties

The average ascorbic acid content was 127.5 mg/100 g wet weight of fresh fruit. The concentration of oxalate averaged 8.214 mg/100 g wet weight of the fruit, while the fiber content averaged 4.659% of wet weight of fruit (Table 1).

**Table 1:** The content of ascorbic acid, oxalate, and fiber in fresh Japanese quince fresh fruit

The parameter studied	Ascorbic acid	Oxalate	Fiber
	[mg/100 g]	[mg/100 g]	[%]
Arithmetic mean ($\overline{x}$)	127.50	8.214	4.659
Standard deviation (±SD)	23.464	0.978	0.497
Maximum	172.00	9.450	5.330
Minimum	100.00	6.670	4.020

Analysis of the microelements showed that the average iron content was 0.516 mg/100 g, copper 0.146 mg/100 g, zinc 0.546 mg/100 g, manganese 0.25 mg/100 g, and molybdenum 0.020 mg/100 g of dry weight (Table 2).

**Table 2:** The content of microelements in Japanese quince fruit

The parameter studied	Fe	Cu	Zn	Mn	Mo
	[mg/100 g dry weight]				
Arithmetic mean ($\overline{x}$)	0.516	0.146	0.546	0.25	0.020
Standard deviation (±SD)	0.165	0.076	0.493	0.013	0.002
Maximum	0.830	0.325	1.456	0.23	0.022
Minimum	0.330	0.093	0.211	0.26	0.018

Analysis of the macro-elements revealed that the average magnesium concentration was 16.729 mg/100 g and calcium 22.920 mg/100 g, phosphorus 64.090 mg/100 g, potassium 249.740 mg/100 g, and sodium 2.805 mg/100 g of dry weight of Japanese quince fruit (Table 3).

**Table 3:** The content of macro-elements in Japanese quince fruit

The parameter studied	Mg	Ca	P	K	Na
	[mg/100 g dry weight]				
Arithmetic mean ($\overline{x}$)	16.729	22.920	64.090	249.740	2.805
Standard deviation (±SD)	3.652	5.687	1.610	2.540	0.183
Maximum	22.835	32.318	65.801	246.090	2.980
Minimum	11.391	17.191	62.071	251.760	2.560

The average dry matter content of the samples obtained by drying was 16.6 g/100 g. The extract content determined by refractometry averaged 9.9%. The acidity of the fresh fruit was 47.50% titratable acidity (as malic acid). Antioxidant activity of fresh fruit averaged 108.00 mM Trolox/g of fresh fruit, and phenolic compounds determined with catechins were 9.96 mg/g of fresh fruit (Table 4).

**Table 4:** The dry weight, the content of the extract, acidity, antioxidant status, and phenolic compounds of Japanese quince fresh fruit

The parameter studied	Dry weight [g/100 g]	Extract [%]	Acidity [%]	Antioxidant activity [mM Trolox/100 g]	Phenolic compounds [mg catechins/100 g]
Arithmetic mean ($\overline{x}$)	16.66	9.899	4.75	1,080,300	996.00
SD	4.40	1.068	0.116	1,591,942	96.171
Maximum	20.1	12.24	4.85	1,278,000	1200
Minimum	11.7	8.78	4.62	800,000	920.00

### The Effect of the Extract of Quince on the Culture of Hepatocytes

**Fig. 1:**
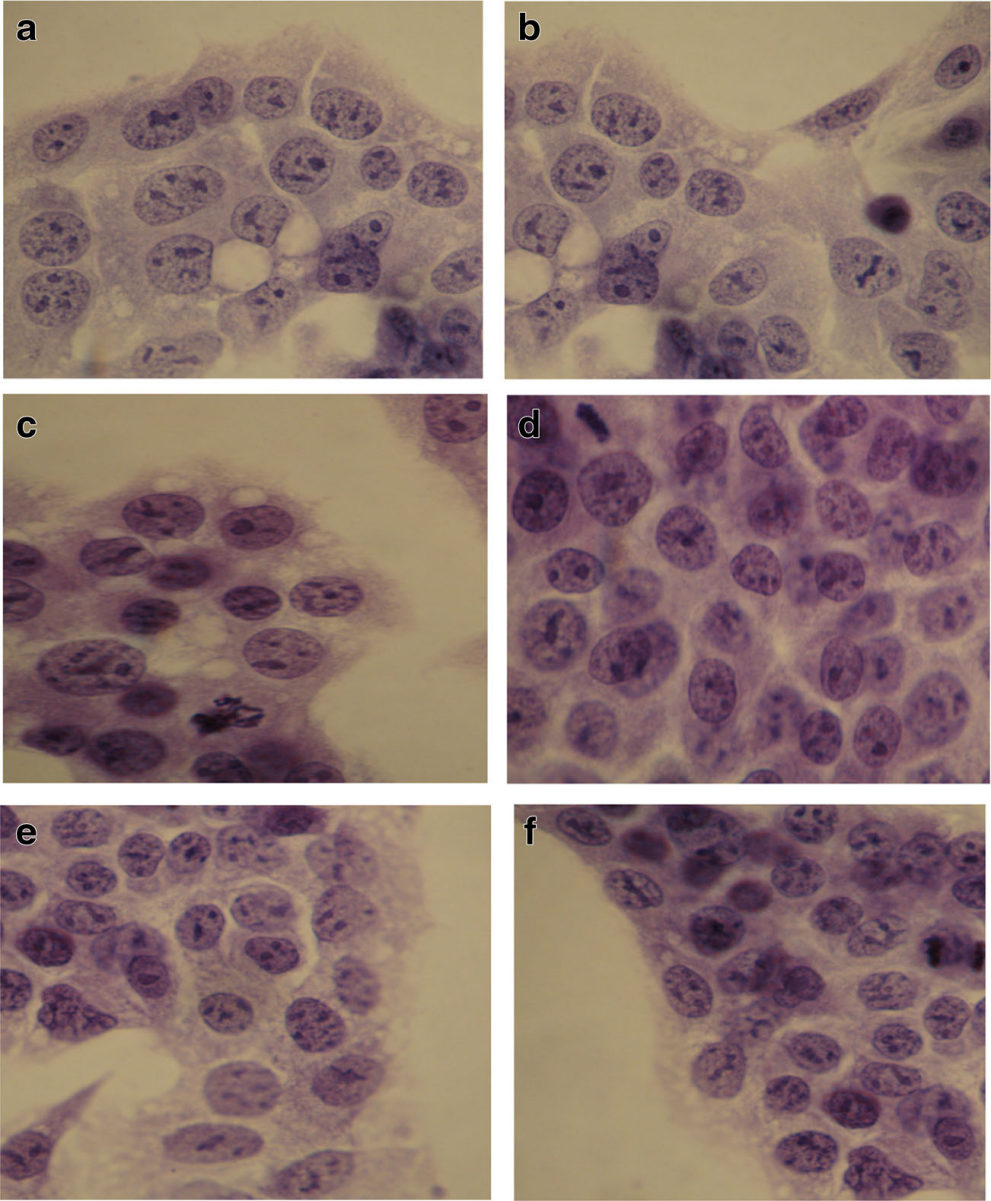
**a**–**f** Cultures of HepG2 cells under light microscopy. The cells were grown under standard test conditions. Acidic hematoxylin staining; lens magnification ×60. **a** Control. **b** Positive control—cells incubated with the addition of medium and 5 mg/mL of DMSO. **c** Cells incubated with 1 μg/mL extract of quince. **d** Cells incubated with 10 μg/mL extract. **e** Cells incubated with 50 μg/mL extract. **f** Cells incubated with 100 μg/mL extract of Japanese quince

No significant differences were seen between the hepatocytes cultured in the control medium nor any of the medium supplemented with an aqueous extract of quince (E), in culture images examined using light microscopy (Fig. 1).

### The Effect of the Extract of Japanese Quince on Serum Lipid Peroxides

**Fig. 2:**
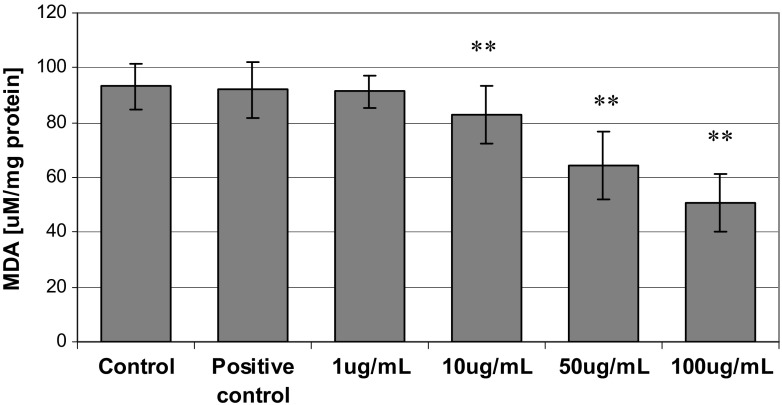
The effect of incubation of hepatocytes with the extract of Japanese quince with increasing concentrations of 1, 10, 50, and 100 μg/mL of malondialdehyde (MDA). The *asterisks* indicate statistically significant difference vs control group (Wilcoxon test)

In hepatocyte cultures incubated with the extract of quince with increasing concentrations of 1, 10, 50, and 100 μg/mL of the extract, MDA was observed to statistically significantly decrease in lipid peroxide concentration compared to the control (*p* = 0.038 for 1 μg/mL; *p* = 0.005 for 10 μg/mL; *p* = 0.02 at 50 μg/mL; *p* = 0.002 for 100 μg/mL of extract). The largest decrease in the concentration of MDA obtained in the trials was from the medium treated with the highest concentration of the extract, 100 μg/mL, at 45%. A proportionally lower decrease (31%) was observed at a concentration of 50 μg/mL. The concentration of 10 μg/mL decreased the concentration of MDA by 11% in relation to the control. MDA was strongly negatively correlated with the concentration of the extract of quince *R* = −0.85, *p* = 0.002 (Fig. 2).

### Effect of the Extract of Japanese Quince on Apoptosis and Necrosis Severity

**Fig. 3:**
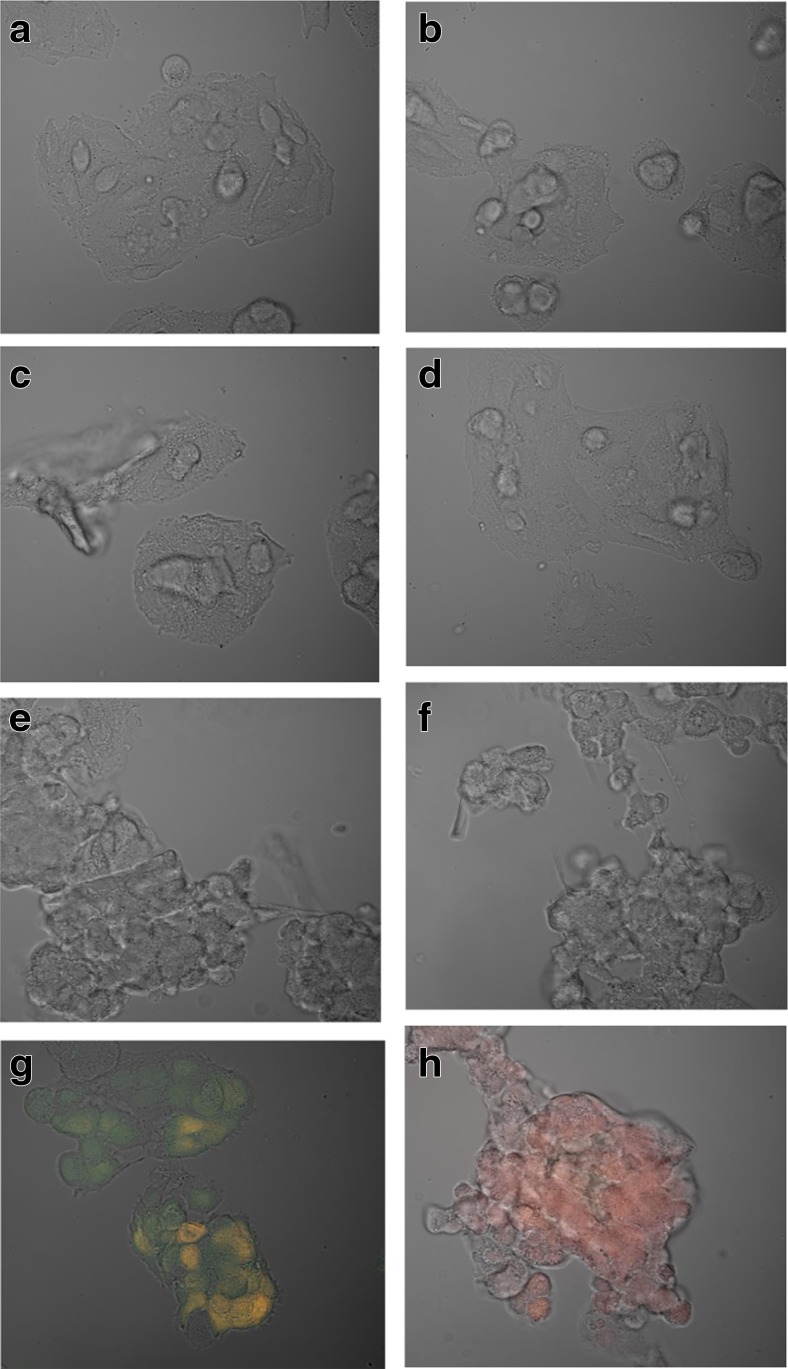
**a**–**h** Evaluation of apoptosis/necrosis. Images from a confocal microscope (FV1000 (Olympus, Germany) showing hepatocytes incubated with Annexin V and propidium iodide (PI), made using a kit of reagents (Annexin V-FITC Early Apoptosis Detection Kit) in the previous 48 h. Cell culture medium with the addition of an extract of quince. **a** Control. **b** A positive control. **c** 1 μg/mL. **d** 10 μg/mL. **e** 50 μg/mL. **f** 100 μg/mL. **g** A positive control for early apoptosis hepatocytes cultured in a medium with 10 μM ethyl alcohol. **h** A positive control of late apoptosis/necrosis-hepatocytes cultured in a medium containing 100 μM ethanol

On the basis of image analysis by confocal microscopy, there was no evidence of apoptosis in hepatocytes incubated with the extract of quince at any concentration used (Fig. 3a). For comparison, hepatocytes were cultured under standard culture conditions with the addition of ethyl alcohol. In hepatocytes incubated with alcohol at 10 μM, there was evidence of early apoptosis—green fluorescence derived from FITC (Fig. 3g)—and late apoptosis/necrosis of hepatocytes incubated with 100 μM alcohol—red fluorescence derived from Annexin V (Fig. 3h). Pictures were taken using a laser: a multiline Ar argon laser (wavelength 488 nm emission, 514/529 nm probe—green fluorescence) and using a HeNe-G helium neon laser (issue 575/625 nm—red fluorescence) (Fig. 3).

### Effect of the Extract of Japanese Quince on the Concentration of Reactive Oxygen Species (ROS) in Hepatocytes

**Fig. 4:**
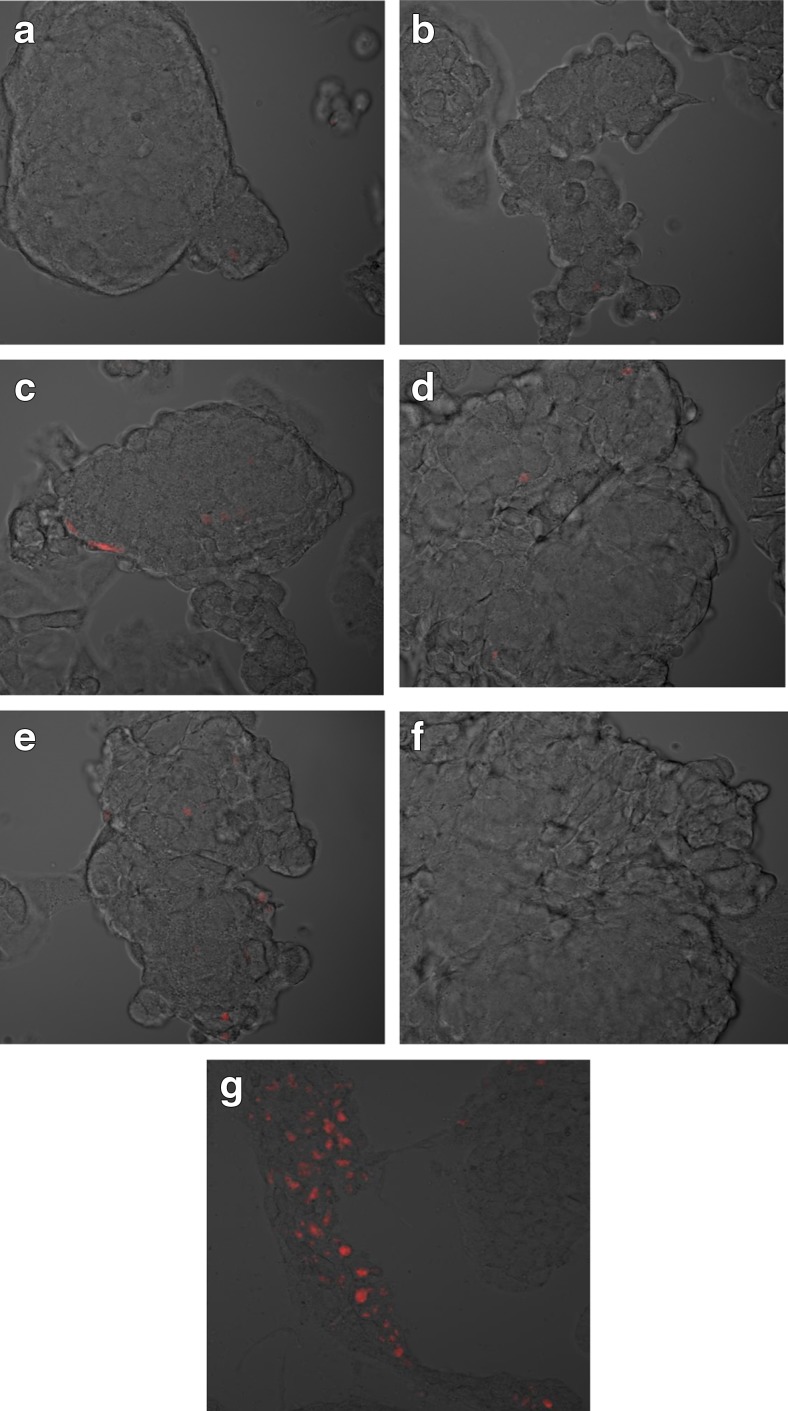
**a**–**g** Intensity of ROS production in mitochondria. Images from a confocal microscope (FV1000 (Olympus, Germany) showing hepatocytes incubated with the probe mitochondrial MitoSOX, after 48 h cultivation of cells in a medium containing an extract of Japanese quince. **a** Control. **b** Positive control. **c** 1 μg/mL. **d** 10 μg/mL. **e** 50 μg/mL. **f** 100 μg/mL. **g** Positive control for enhanced ROS formation—hepatocytes cultured in a medium supplemented with 10 μM of ethyl alcohol. Pictures were taken using a HeNe-G helium neon laser (575/625 nm wavelength—*red* fluorescence) (color figure online)

On the basis of image analysis by confocal microscopy, no evidence was seen of increased ROS formation in the mitochondria of hepatocytes incubated with the extract of quince at any concentration used (Fig. 4a, f). For comparison, hepatocytes were cultured under standard culture conditions with the addition of ethyl alcohol. The hepatocytes incubated with alcohol at 10 μM showed evidence of increased ROS formation, appearing as red fluorescence from the probe (MitoSOX) (Fig. 4g).

## Discussion

The presented results show the specific characteristics of Japanese quince (*C. japonica*) fruit properties. Our analysis showed the fruit of *C. japonica* had high levels of macro- and microelements, ascorbic acid, phenolic compounds, fiber, and low oxalate levels. The work by Lesińska [10] also indicates that the Japanese quince fruit is a valuable source of minerals, particularly of Mg, Fe, Zn, and Cu (Table 5). The quite substantial content of ascorbic acid (55–92 mg/100 g) and its stability during storage and processing make it a good, natural source of vitamin C. In the studied fruit, the content of the ascorbic acid was nearly twice as high as in the study by Lesińska [10] and Hallmann et al. [4] (Table 5). In the pome accessory fruit group, the fruit of Japanese quince may be considered as the biggest source of minerals. Despite these desirable characteristics, the Japanese quince group is also highly acidic, making the plants inappropriate for raw consumption. However, very high acidity makes the half products of Japanese quince valuable as additions to mixed products because they make them more acidic in a natural way and enrich them with several valuable qualities. The study by Nawirska-Olszańska et al. [15, 16] evaluated the quality of jams made from pumpkin with the addition of strawberry, cornelian cherry, and Japanese quince. It was observed that jams in which the Japanese quince constituted 30 and 50% of weight had the highest contents of ascorbic acid, 10.39 mg/100 g and 9.24 mg/100 g, respectively. The polyphenol content was also the highest in jams with the addition of *Chaenomeles* fruit, ranging from 0.257 to 0.945 mg of gallic acid per gram.

**Table 5:** The comparison of Japanese quince fruit properties based on literature data

Parameters	[29, 30]	[4]	[10]
Ascorbic acid [mg/100 g fresh fruit]	–	62.7 ± 10.5	68.8 ± 7.71
Fiber [g]	1.35 ± 0.05	–	3.88 ± 0.64
Dry weight [%]	12.9 ± 0.43	12.8 ± 0.2	15.37 ± 0.38
Extract [%]	9.4 ± 0.72	–	10.1 ± 0.63
Acidity [%]	4.1 ± 0.02	–	3.70 ± 0.19
Antioxidant activity [μM Trolox/100 g]	10,512.0 ± 37.0	–	–
Phenolic compounds [mg catechins/100 g]	924.0 ± 11.71	–	–
Fe [mg/100 g fresh fruit]	–	–	1.8
Cu [mg/100 g fresh fruit]	–	–	0.27
Zn [mg/100 g fresh fruit]	–	–	0.39
Mn [mg/100 g fresh fruit]			0.19
Mo [mg/100 g fresh fruit]			0.046
Mg [mg/100 g fresh fruit]	–	–	14.0
Ca [mg/100 g fresh fruit]	–	–	23.3
K [mg/100 g fresh fruit]	–	–	253
Na [mg/100 g fresh fruit]	–	–	3.6

The analysis of fresh Japanese quince fruit and the cornelian cherry fruit carried out by Tarko et al. [29] showed higher dry matter content in dogwood/cornelian cherry fruit, i.e., 20.31% in comparison to the 12.89% in the Japanese quince fruit. The two analyzed kinds of fruit had a similar amount of fiber, 1.35% in Japanese quince and 1.53% in cornelian cherry. The acidity of the Japanese quince fruit, calculated as malic acid/titratable acidity [%], was higher than the acidity of the cornelian cherry, 4.11 in comparison to 3.91%. Furthermore, in comparison to the cornelian cherry, the fruit of the Japanese quince expressed a higher antioxidative activity (10,512 and 7123 μM Trolox/100 g, respectively) and a higher content of polyphenols (924 and 611 mg catechin/100 g, respectively). Other studies performed by Tarko et al. [30] that compared the suitability of the Japanese quince, cornelian cherry, and black mulberry fruit as raw materials for processing confirm the high acidity of the first two types of fruit and the similarly high antioxidant activity and total phenolic content of all the three analyzed types of fruit. Further studies by Tarko et al. [31] concerning the use of fruit extracts for the production of beverages with high antioxidative activity or the use of fruit extracts for beverage enrichment in order to increase antioxidative potential and polyphenol content revealed that the addition of extracts from the Japanese quince fruit to the apple and orange beverages and the addition of lingonberry extract to the grapefruit beverage were the preferred choices in comparison to the extracts from the cornelian cherry, elderberry, and hawthorn. For example, the 5% addition of the Japanese quince extract was associated with the increased antioxidative activity and polyphenol content in the orange beverage (by 24 and 56%, respectively).

The work by Fronc and Oszmiański [3] analyzed the suitability of Japanese quince and chokeberry for the production of infusions: the content of total polyphenols in Japanese quince was 6.45 mg/g. Zadernowski et al. [41] also conducted research on the content of phenolic compounds in stone fruits. Among the tested fruits (apples, pears, quince, Japanese quince, nectarines, plums, blackthorn, cherry, peach, and American cherry), the highest amount of phenolic compounds was demonstrated in the Japanese quince with 43 g/kg dry mass, which corresponded to 4.9 g/kg of fresh fruit. The Japanese quince fruit also showed the highest content of tannins (0.3 g/kg of fresh fruit). Our research confirms the high content of phenolic compounds and high antioxidant activity of the Japanese quince.

Wojdyło et al. [40] also showed that the Japanese quince fruit is rich in fiber, simple sugars, as well as compounds from the polyphenol group: catechin, epicatechin, and procyanidins. Hallmann et al. [4] showed that the content of dry matter in the Japanese quince was 12.8 ± 0.2 g/100 g, vitamin C: 62.7 ± 10.2 mg/100 g fresh fruit. The content of total phenolic acids was 2.2 mg/100 g fresh fruit and total flavonoids were 17.1 mg/100 g fresh fruit. The 100 g of fresh fruit of the Japanese quince contained 2.2 ± 0.3 of milligram *p*-coumaric per 100 g fresh fruit, 2.23 ± 0.1 mg of kaempferol d-glucoside, 2.94 ± 0.4 mg 3-quercetin rutinoside, and 11.9 ± 0.2 mg of myricetin [4]. The results are similar to those presented by Tang et al. [32], where the total phenolic content in other flowering quince *C. speciosa* fruit was 50 mg/100 g fresh fruit.

Zhang et al. [43] showed other *Chaenomeles* species (*C* *haenomeles lagenaria* syn*. C. speciosa*) contained not only phenolic and phenylpropionic acids but also triterpenoids, flavonoids, saccharides, essential oils, and alkaloids. Triterpenoid acids, oleanolic and ursolic acid in particular, are the major active constituents that possess several pharmacological properties in vivo and in vitro, including anti-inflammatory and hepatoprotective properties [38].

Also, our results clearly show the positive effect of aqueous extract of the fruit of *C. japonica* on hepatocytes. The addition of an aqueous extract of Japanese quince did not result in any negative changes to the images of hepatocytes cultures under light microscopy (Fig. 1). In contrast, in the tested hepatocyte cultures incubated with extract of the Japanese quince, we observed a significant decrease in the concentration of lipid peroxides compared to the control. The greatest reduction in MDA concentration was observed at the highest concentration of extract (a 45% decrease relative to the control using an extract concentration of 100 μg/mL). There were also no signs of increased formation of reactive oxygen species in the mitochondria of hepatocytes incubated with the extract of quince. MDA was strongly negatively correlated with the concentration of Japanese quince extract, which indicates the hepatoprotective properties of Japanese quince, i.e., a reduction in the concentration of reactive oxygen species. In addition, our analysis of confocal microscopy images showed that the hepatocytes incubated with the extract of Japanese quince at any concentration did not show any signs of apoptosis or necrosis. This study suggests that the use of extracts of the Japanese quince does not entail negative consequences for hepatocytes and instead has a hepatoprotective effect.

## Conclusions

A characteristic feature of the fresh fruit of *C. japonica* is the high content of macro- and microelements and high levels of polyphenols, which—combined with a high content of vitamin C—affect their high antioxidant potential. The aqueous extract of quince fruit has antioxidative and antiapoptotic hepatocytes, thus exerting a hepatoprotective effect.
